# A new genus of oak gallwasps, *Zapatella* Pujade-Villar & Melika, gen. n., with a description of two new species from the Neotropics (Hymenoptera, Cynipidae, Cynipini)

**DOI:** 10.3897/zookeys.210.3014

**Published:** 2012-07-24

**Authors:** Juli Pujade-Villar, Paul Hanson, Claudia A. Medina, Miguel Torres, George Melika

**Affiliations:** 1 Universitat de Barcelona, Facultat de Biologia, Departament de Biologia Animal, Avda. Diagonal 645, 08028-Barcelona, Spain; 2 Universidad de Costa Rica. Escuela de Biologia. San Pedro, Costa Rica; 3 Instituto de Investigación de Recursos Biológicos Alexander von Humboldt, Claustro de San Agustín, Villa de Leyva, Colombia; 4 Pest Diagnostic Laboratory, Plant Protection & Soil Conservation Directorate of County Vas, Ambrozy setany 2, Tanakajd 9762, Hungary

**Keywords:** Cynipini, *Zapatella*, *Callirhytis*, Colombia, Costa Rica, taxonomy, morphology, distribution, biology

## Abstract

A new genus of cynipid oak gallwasp, *Zapatella* Pujade-Villar & Melika, **gen. n.** (Hymenoptera: Cynipidae: Cynipini), with two new species, *Zapatella grahami* Pujade-Villar & Melika, **sp. n.** and *Zapatella nievesaldreyi* Melika & Pujade-Villar, **sp. n.**, is described from the Neotropics. *Zapatella grahami*,known only from the sexual generation,induces galls in acorns of *Quercus costaricensis* and is currently known only from Costa Rica. *Zapatella nievesaldreyi*, known only from the asexual generation, induces inconspicuous galls in twigs of *Quercus humboldtii*, and is known only from Colombia. Diagnostic characters for both new species are given in detail. Five Nearctic species are transferred from *Callirhytis* to *Zapatella*: *Zapatella cryptica* (Weld), **comb. n.**, *Zapatella herberti* (Weld), **comb. n.**, *Zapatella oblata* (Weld), **comb. n.**, *Zapatella quercusmedullae* (Ashmead), **comb. n.,** *Zapatella quercusphellos* (Osten Sacken), **comb. n.** (= *Zapatella quercussimilis* (Bassett), **syn. n.**). A key based on adults for the species belonging to *Zapatella* is also given. Generic limits and morphological characteristics of *Zapatella* and closely related genera are discussed.

## Introduction

The cynipid gallwasp fauna (Hymenoptera, Cynipidae) of the Neotropical region is very poorly known. Recently it was updated to include 6 tribes, 21 genera and 45 species, of which 41 are native and 4 have been introduced into the region; the native fauna includes 17 described species of oak gallwasps and 15 associated inquiline species (Medianero and Nieves-Aldrey 2011b). The Neotropics, particularly southern Mexico, harbours the greatest diversity of oak species in the New World. Some species are widespread from Mexico to Costa Rica and Panama. At least one clade of red oaks (Section Lobatae of *Quercus* subg. *Quercus* L.) is common in Central America and Colombia (Govaerts and Frodin 1998, Nixon 2006). In Mexico, gallwasps, gallwasps were reported from 11 different red oak species (Pujade-Villar et al. 2009; Melika et al. 2009a, 2011a; Pujade-Villar et al. 2011), and on all oaks 157 gallwasp species on 33 oak species are mentioned (Pujade-Villar et al. 2009). A large diversity of oak gallwasps is also known from Panama, where 65 different cynipid galls were found on oaks, and from 45 of these galls, adult gallwasps were reared: *Andricus* Hartig (12 species), *Neuroterus* Hartig (9), *Dryocosmus* Giraud (7), *Cynips* L. (4), *Amphibolips* Reinhard (3), *Disholcaspis* Dalla Torre & Kieffer, *Loxaulus* Mayrand *Odontocynips* Kieffer (with two species each), *Callirhytis* Förster and *Bassettia* Ashmead (with one species each) (Medianero and Nieves-Aldrey 2011b). Ten of these species have been described (Medianero and Nieves-Aldrey 2010a, b, 2011a, Medianero et al. 2011a, b). Thus far, only four oak gallwasp species have been described from Costa Rica: *Odontocynips hansoni* (Pujade-Villar 2009), *Andricus costaricensis* (Melika et al. 2009b), *Disholcaspis costaricensis* (Melika et al. 2011b) and *Coffeikkokos copeyensis* (Pujade-Villar et al. 2012); however, there might be more than 30 species in total (Fergusson 1995, Pujade-Villar and Hanson 2006). Another six species are known from Guatemala (Cameron 1883, Kinsey 1936, Weld 1952) and one species from the southern part of Mexico (Kinsey 1937).

The evaluation of the Neotropic gallwasp fauna cannot be done without a thorough examination of the Nearctic species, especially in the case of establishing new gallwasp genera. The current morphology-based taxonomy of the Nearctic Cynipini, with the last review of genera by Melika and Abrahamson (2002), needs a major revision at both the genus and species levels. Sets of character states used to identify genera/species are often not appreciable for taxonomic purposes; in many genera plesiomorphic characters were used instead of synapomorphies or autapomorphies, and thus the majority of current Cynipini genera in the Nearctic are polyphyletic, instead of being monophyletic. The new data recently obtained on the phylogeny, phylogeography, evolutionary conservatism of host shifts were not considered in the previous reviews and revisions (Liljeblad et al. 2008, Stone et al. 2009).

The validity of some Nearctic species of *Callirhytis* and there taxonomic position are discussed.

## Material and methods

Adult gallwasps of an undescribed species were reared from acorn galls collected on *Quercus costaricensis* by the second author (PH) in Costa Rica; specimens belonging to yet another species were reared from galls collected on *Quercus humboldtii* by the first author (JPV) together with Claudia A. Medina and Miguel Torres in Colombia.

We follow the current terminology of morphological structures (Liljeblad and Ronquist 1998, Melika 2006). Abbreviations for forewing venation follow Ronquist and Nordlander (1989), cuticular surface terminology follows that of Harris (1979). Measurements and abbreviations used here include: F1–F12, 1st and subsequent flagellomeres; POL (post-ocellar line) is the distance between the inner margins of the posterior ocelli; OOL (ocellar-ocular line) is the distance from the outer edge of a posterior ocellus to the inner margin of the compound eye; LOL (lateral ocellar line), the distance between lateral and frontal ocelli. The width of the forewing radial cell is measured from the margin of the wing to the Rs vein.

Digital images of wasp anatomy were produced with a digital Nikon Coolpix 4500 camera attached to a Leica DMLB compound microscope, followed by processing in CombineZP (Alan Hadley) and Adobe Photoshop 6.0 by the last author (GM). The SEM pictures were taken with a Stereoscan Leica-360 by Palmira Ros-Farré (Barcelona University, Spain) at a low voltage (15KV) and with gold coating; the forewing of *Zapatella nievesaldreyi* was taken by JPV with a digital camera Cannon SX-210-IS, attached directly to the ocular of a stereomicroscope. Gall images of *Zapatella grahami* were taken by P. Hanson; galls of *Zapatella nievesaldreyi* by the fourth author (M T).

The type material is deposited in the following institutions:

**UB** University of Barcelona, Spain (J. Pujade-Villar);

**PDL** Pest Diagnostic Laboratory (the former Systematic Parasitoid Laboratory, SPL), Tanakajd, Hungary (G. Melika);

**MZUCR** Museum of Zoology, University of Costa Rica, San Pedro Costa Rica (Paul Hanson);

**IAvH** Instituto Alexander von Humboldt, Villa de Leyva, Colombia (Claudia Medina).

## Results

### Zapatella

Pujade-Villar & Melika gen. n.

urn:lsid:zoobank.org:act:D093C259-5DB1-43AE-A999-0AD53B8F4EA4

http://species-id.net/wiki/Zapatella

Figures 1 [Fig F2] [Fig F3] [Fig F4] [Fig F5] [Fig F6] [Fig F7] [Fig F8] [Fig F9] 62

#### Type species.

*Zapatella grahami* Pujade-Villar & Melika, sp. n. by present designation.

#### Diagnosis.

Partially resembles *Callirhytis*, *Bassettia* and *Plagiotrochus*. However, in *Zapatella*, the malar sulcus is absent; mesosoma strongly arched, short, as long as high in lateral view; mesoscutum with numerous fine short, interrupted transverse striae with numerous longitudinal anastomosis connecting transverse striae and together forming a net-like, delicately reticulate, irregular sculpture; the pronotum laterally delicately reticulate; the metascutellum rugoso-reticulate; the metanotal trough and the lateral area of the propodeum with dense white setae. In *Callirhytis* a distinct malar sulcus is present; the mesosoma less arched, always at least slightly longer than high in lateral view; the transversely orientated rugae on the mesoscutum are much stronger with much fewer anastomoses between them; the pronotum with distinct strong rugae laterally; the metascutellum rugose, never reticulate; the metanotal trough and the lateral area of the propodeum without or with very few setae. In *Bassettia* the mesosoma is strongly compressed dorsolaterally, distinctly longer than broad; the head always more massive from above and nearly rounded in anterior view, broader than the mesosoma. In *Plagiotrochus* the sculpture of the mesopleuron, the shape of propodeal carinae and the length of the prominent part of the ventral spine of the hypopygium are quite different. The most striking characters that differentiates *Zapatella* from the above-mentioned genera are the long prominent part of the ventral spine of the hypopygium, which is 6.0–8.5 times longer than broad; hind coxae with dense white setae on the dorsoposterior surface, while in the other mentioned genera the prominent part of the ventral spine of the hypopygium is very short, at most 2–3 times longer than broad, and hind coxae without dense setae. For more details see also the Discussion.

**Figures 1–8.**
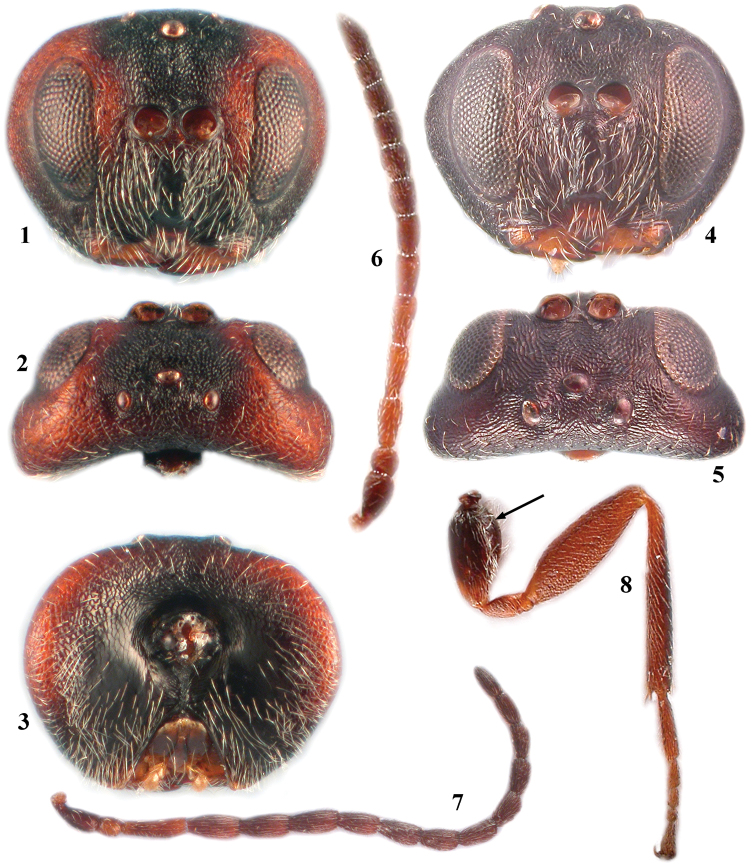
*Zapatella grahami* **1** head, female (anterior view) **2** head, female (dorsal view) **3** head, female (posterior view) **4** head, male (anterior view) **5** head, male (dorsal view) **6** antenna, female **7** antenna, male **8** hind leg, female (arrow indicates the dense white setae on dorsoposterior surface of coxa).

#### Description.

Body, including antennae and legs, predominantly chestnut brown; in some species head partially, mesoscutellum and stripes on mesoscutum dark brown to black. Head 1.3–1.5 times as broad as high in anterior view, massive from above and slightly broader than mesosoma. Gena broadened behind eye, as broad as transverse diameter of eye; malar sulcus absent. Antenna with 11 flagellomeres in female, 13 in male.

Mesosoma strongly arched, short, as long as high in lateral view. Pronotum delicately reticulate laterally; mesoscutum with numerous fine interrupted short transverse striae with numerous longitudinal anastomosis connecting transverse striae and together forming a net-like, delicately reticulate, irregular sculpture. Notauli complete (only in *Zapatella herberti*) or incomplete, extending to 1/2–2/3 length of mesoscutum, converging, deep and broad posteriorly [in some species, on first view, notauli seem to be complete; however, these are just darker lines, not impressed notauli, e.g. *Zapatella quercusmedullae*]. Anterior parallel lines extending to 1/2 length of mesoscutum; parapsidal lines distinct and broad, starting from posterior margin and extending to 1/2 length of mesoscutum; median mesoscutal line present or absent. Mesoscutellum 0.5 times as long as mesoscutum, as long as broad, not or only slightly overhanging metanotum, center of disk reticulate, sides and posterior 1/3–2/3 dull rugose; scutellar foveae present, indistinctly delimited posteriorly. Mesopleuron uniformly delicately reticulate, smooth and shiny basally. Metascutellum rugoso-reticulate; metanotal trough and lateral propodeal area with dense setae. Central propodeal area delimited by distinct subparallel or slightly bented outwards lateral propodeal carinae. Dorsoposterior surface of hind coxa with dense white setae. Tarsal claws simple, without basal lobe. Forewing venation pale yellow, indistinct, R1 inconspicuous, hardly traceable; wing margin without cilia. 2nd metasomal tergite with felt-like dense ring of white setae, interrupted dorsally and few setae scattered on lateral surface of tergite; narrow posterior band on 2^nd^ metasomal tergite and all subsequent tergites with very delicate dense micropunctures. Prominent part of ventral spine of hypopygium very long, 6.0–8.5 times longer than broad, with very few short white setae in two rows, directed ventrally; subapical setae absent.

#### Etymology.

Based on a word-play in football, a joke often used between some coauthors and prof. Graham N. Stone (Edinburgh University), in honour of whom one of the species is named.

#### Gender.

Feminine.

#### Biology.

According to the emergence dates of adults obtained from the collected galls, both sexual and asexual forms are present in the newly described genus. However, the emergence periods of alternate generations are overlapping. Moreover, no morphological differences have been observed between sexual and asexual females. The duration of life cycle is probably more than one year. In the Neotropical area the sexual form (*Zapatella grahami* Pujade-Villar & Melika, sp. n.)is obtained from acorn galls, while the asexual form (*Zapatella nievesaldreyi* Melika & Pujade-Villar,sp. n.) from twig galls; in the Nearctic area the asexual forms are obtained from twig and bud galls (*Zapatella cryptica* (Weld), comb. n., *Zapatella herberti* (Weld), comb. n., *Zapatella quercusmedullae* (Ashmead), comb. n.), *Zapatella oblata* (Weld), comb. n.,while the sexual form, *Zapatella quercusphellos* (Osten Sacken) comb. n.(*= quercussimilis* (Bassett), syn. n. from twig galls. A detailed study of the biological cycles is necessary to solve this problem, which might be partially similar to that found in *Plagiotrochus amenti* Kieffer whichhas two reproductive modes: a heterogonic life cycle with alternation of generations in the circum-Mediterranean region, and an asexual, parthenogenetic life cycle in North America (Garbín et al. 2008), but the most important aspect is that in the Mediterranean area *Plagiotrochus amenti* has a partially overlapping emergence of the asexual and sexual forms (Benia et al. 2009). The same heterogenetic life cycle was also found in another Western Palaearctic gallwasp, *Andricus quadrilineatus* Hartig (Folliot 1961, 1964).

#### Distribution.

Currently known fromthe Neotropics(Costa Rica and Colombia) and the Nearctic (USA, from California, through Texas to Florida and along the Atlantic coast, up to New York state), after transferring 4 *Callirhytis* species.

### Zapatella grahami

Pujade-Villar & Melika sp. n.

urn:lsid:zoobank.org:act:B802BBE3-E4BF-4971-9569-96266500DBE4

http://species-id.net/wiki/Zapatella_grahami

#### Type material.

HOLOTYPE female (deposited in UB): “COSTA RICA, Cartago-Jose, Cerro de la Muerte, 3000 m, 2.X.1988. Col. Hanson” (white label), “*Quercus costaricensis*, fruit (acorn) galls” (white label), Holotype of *Zapatella grahami* ♀ Pujade-Villar & Melika n. gen & n. sp. design. JP-V 2012” (red label). PARATYPES (5 males and 20 females): 3 males and 14 females with the same data as the holotype and 1 male and 1 female with the similar data, only the collecting date is II.1988. 2 males and 10 females are deposited in UB, 1 male and 5 females in PDL, 1 male and 3 females in MZUCR, 1 male and 2 females USNM.

#### Diagnosis.

In *Zapatella* three species, *Zapatella oblata*, *Zapatella grahami* sp. n. and *Zapatella nievesaldreyi* sp. n., have the head and mesosoma partially dark brown to black. *Zapatella oblata* differs from the two other mentioned species by a very long median mesoscutal line which extending to 2/3 of the mesoscutum length, while in *Zapatella grahami* and *Zapatella nievesaldreyi* the median mesoscutal line is absent or present in a form of a very short triangle. In *Zapatella grahami* the females are much darker, POL 1.4 times as broad as OOL (Fig. 2), bottom of scutellar foveae with rugae (Fig. 11), and the prominent part of the ventral spine of the hypopygium 7.5–8.5 times as long as broad (Figs 14, 16). In *Zapatella nievesaldreyi* the females are lighter, POL equal OOL (Fig. 20), bottom of scutellar foveae smooth and without rugae (Fig. 22), and the prominent part of the ventral spine of the hypopygium 6.0–7.0 times as long as broad (Figs 28–29).

**Figures 9–13. F2:**
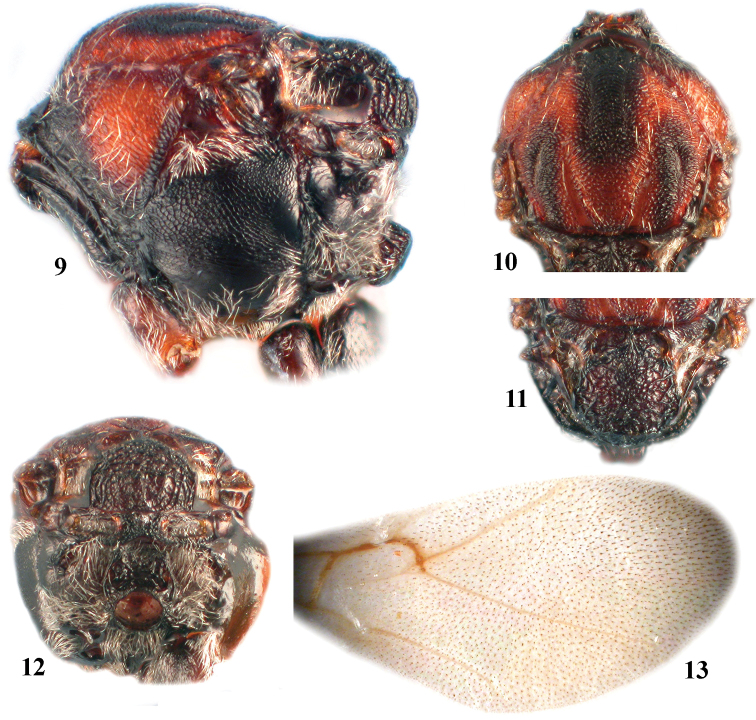
*Zapatella grahami*,female **9** mesosoma (lateral view) **10** mesoscutum (dorsal view) **11** mesoscutellum (dorsal view) **12** metascutellum and propodeum (posterodorsal view) **13** forewing.

#### Description.

Female (Figs 1–3, 6, 8, 9–16).

**Length.** 2.6–3.2 mm (n=15).

**Coloration.** Body, including antennae and legs predominantly dark to chestnut brown. Head, brown with more or less extensive black areas on lower face, basal part of genae, central part of frons and vertex; posteriorly head dark brown to black. Antenna uniformly dark brown, F1–F5 lighter; mesosoma laterally black, except brown dorsolateral area of pronotum; propleura black; mesoscutum brown, with black stripes along anterior parallel and parapsidal lines; mesoscutellum dark brown to black, with slightly lighter scutellar foveae. Propodeum uniformly black; axillula yellowish; legs uniformly brown, with darker hind legs; metasoma brown, anterodorsally darker.

**Head** (Figs 1–3). Uniformly and delicately reticulated, with few white setae, 1.8–2.0 times as broad as long from above, 1.3–1.5 times as broad as high in frontal view and slightly broader than mesosoma. Gena broadened behind eye, as broad as transverse diameter of eye; malar space 0.35–0.4 times as long as height of eye, with delicate striae radiating from clypeus and nearly reaching eye margin, malar sulcus absent. POL 1.4 times as long as OOL; OOL 2.5 times as long as length of lateral ocellus and 1.8 times as long as LOL. Transfacial distance nearly 1.2 times as broad as height of eye; diameter of antennal torulus around 3.8 times as great as distance between them, distance between torulus and inner margin of eye equal to or slightly longer than diameter of torulus; inner margins of eyes parallel; lower face delicately coriaceous, with dense white setae, the median elevated area smooth. Clypeus small, squared, smooth, impressed in basal part, ventrally straight; anterior tentorial pits, epistomal sulcus and clypeo-pleurostomal line indistinct. Frons, vertex, interocellar area and occiput delicately reticulate. Postocciput alutaceous and shiny, smooth and impressed around occipital foramen; posterior tentorial pits large; height of occipital foramen as long as height of postgenal bridge; hypostomal carina emarginate, not going around oral foramen, continuing into gular sulcus. Labial palpus 3-segmented, terminal peg distinct, all three segments densely setose; maxillary palpus 5-segmented, terminal peg distinct, three terminal segments densely setose.

**Figures 14–18. F3:**
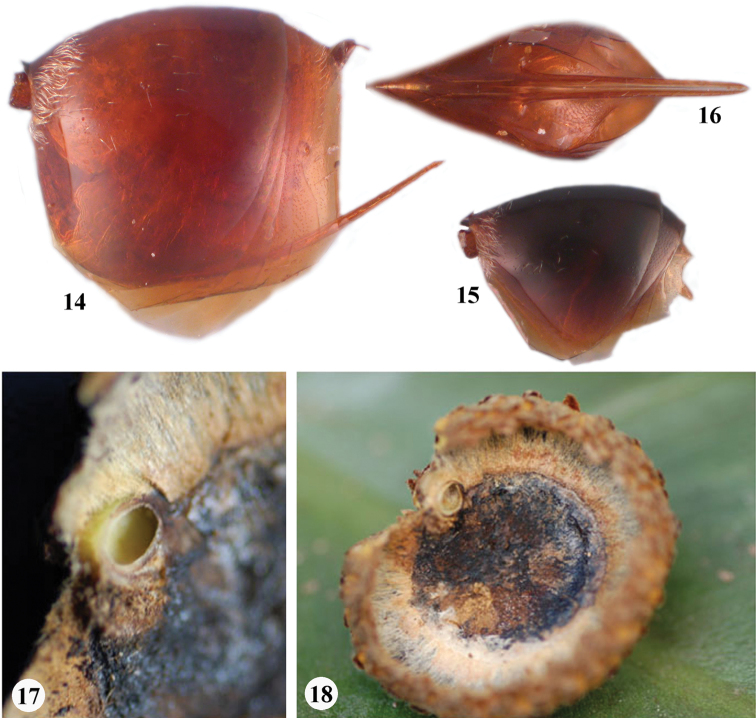
*Zapatella grahami* **14** metasoma, female (lateral view) **15** metasoma, male (lateral view) **16** ventral spine of hypopygium (ventral view) **17–18** gall.

**Antenna** (Fig. 6). With 11 flagellomeres (14: 9×8: 15×8: 19: 15: 14: 13: 11: 10: 10: 9: 8: 16); longer than head+mesosoma (48:34); pedicel globose, as long as broad; F1 as long as scapus; F2 1.2–1.3 times as long as F1; F1=F3; F4–F5 subequal and shorter, F6–F10 shorter and progressively shortening in length; F11 twice as long as F10; placodeal sensilla distinct on F6–F11, indistinct but present on F4–F5, absent on F1–F3.

**Mesosoma** (Figs 9–12). 1.2–1.3 times as long as high in lateral view, with few white setae. Mesoscutum as long as broad, or only slightly longer than broad in dorsal view; with sparse scattered setae and transverse, delicate, interrupted striae which connect with longitudinally orientated weak striae, forming an irregular network of striae, and an irregularly reticulate surface sculpture. Notauli incomplete, extending at most to half length of mesoscutum; converging, deep and broad posteriorly. Anterior parallel lines extending to ½ length of mesoscutum; parapsidal lines distinct and broad, starting from posterior margin and extending to ½ length of mesoscutum; median mesoscutal line very short or absent. Mesoscutellum 0.5 times as long as mesoscutum, as long as broad, not overhanging metanotum, center of disk reticulate, sides and posterior 1/3 dull rugose; scutellar foveae present, ovate, not delimited posteriorly, bottom shiny, with some rugae; median carina broad. Mesopleuron uniformly delicately reticulate, smooth and shiny basally; mesopleural triangle conspicuously setose; dorsal axillar area coriaceous with numerous setae, lateral axillar area reticulo-carinate, without setae; axillula smooth, with white setae; subaxillular bar smooth, shiny, narrower than height of metanotal trough; postalar process long, strong, reticulate; metapleural sulcus reaching mesopleuron in upper 2/3 of its height. Metascutellum strongly reticulate, rectangular. Metanotal trough with short white setae; ventral impressed area at least twice as narrow as height of metascutellum, delicately reticulate. Propodeum setose lateraly, glabrous centrally; central propodeal area smooth, shiny, with many irregular wrinkles and rugae, lateral propodeal carinae weak, diverging anteriorly and converging in posterior 1/3. Nucha with irregular wrinkles and rugae.

**Legs** (Fig. 8). Tarsal claws simple, without basal lobe; hind coxae with dense white setae on the dorsoposterior surface.

**Forewing** (Fig. 13). Longer than body, hyaline, without cilia on margin; radial cell 3.3 times as long as broad; 2r distinct; R1 absent or hardly visible, Rs very inconspicuous, nearly straight; areolet absent or very indistinct. Rs+M indistinct, reaching basalis at half of its height.

**Metasoma** (Figs 14, 16). Shorter than head+mesosoma, slightly higher than long in lateral view; base of 2nd metasomal tergite with felt-like dense ring of white setae, interrupted dorsally and few setae scattered on lateral surface of tergite. Narrow posterior band on 2^nd^ metasomal tergite and all subsequent tergites with very delicate dense micropunctures. Prominent part of ventral spine of hypopygium needle-like, tapering to apex, 7.5–8.5 times as long as broad, with two parallel rows of short white scattered setae which do not extend beyond the apex of spine.

**Male** (Figs 4–5, 7, 15). Length 2.3–2.5 mm (n=4). Similar to female, except in the following characters: predominantly black with few brown areas; head 2.0 times as broad as long from above, 1.2 times as broad as high and broader than mesosoma in frontal view; malar space 0.3 times as long as height of eye; POL 2.0 times as broad as OOL; OOL 2.0 times as long as length of lateral ocellus and 1.3 times as long as LOL. Antennae with 13 flagellomeres (6: 4×4: 11×3.5: 10: 9: 9: 8: 8: 7: 7: 6: 6: 6: 6: 7); longer than body (101:93); pedicel as long as broad; F1 slightly longer than F2, distinctly curved, dorsally flattened and excavate; subsequent flagellomeres progressively shorter in length; F13 longer than F12; placodeal sensilla on all flagellomeres.

**Gall** (Figs 17–18). Acorn galls. Individual chambers located in the acorn cup, often between the cup and the seed. Usually there is one gall per acorn, but sometime two or three.

**Biology.** Only the sexual generation is known and it induces galls on *Quercus costaricensis*. Galls were collected in February and later in October in forests located above 3000 m altitude, adults emerged immediately after the galls were collected, in February and October. This very unusual emergence of adults in two periods may be due to the sporadic nature of the collecting and to the peculiar phenology of *Quercus costaricensis*. In the area where the galls were collected, Camacho and Orozco (1998) observed the flowering and fruiting phenology for a four year period (July 1986 to July 1990. The female flowers were present for ten months of the year, starting in the rainy season, with a flowering peak in the dry season. Male flowers were present for seven months, with a flowering peak from October to January, the period from the end of the rainy season and continuing to the beginning of the dry season. During the four years of observation there was only one fruiting period, which was synchronous, very productive, and extended for eight months (August 1988 to March 1989); this is one year after the initial production of female flowers and six months after the end of male flower production.

#### Distribution.

Currently known only from Costa Rica (Cerro de la Muerte).

#### Etymology.

In recognition of the continuing contribution of our friend, prof. Graham N. Stone (Institute of Evolutionary Biology, University of Edinburgh, Edinburgh, Scotland) to research on oak gallwasps.

### Zapatella nievesaldreyi

Melika & Pujade-Villar sp. n.

urn:lsid:zoobank.org:act:7D783313-C344-41D4-BD4A-1D2E3E6EE4BB

http://species-id.net/wiki/Zapatella_nievesaldreyi

#### Type material.

HOLOTYPE female (deposited in IAvH): “COLOMBIA, Boyacá, Villa de Leyva, Vereda sabana, Sector Chaina,, 05°41'05.1"N, 73°29'17.3"W, 2468 m. En Agallas en ramas de *Quercus humboldti*, (13 May 2010) May-2010. leg. J. Pujade-Villar, C. Medina, M. Torres” (white label), Holotype of *Zapatella nievesaldreyi* ♀ Melika & Pujade-Villar n. sp. design. JP-V 2012” (red label). PARATYPES (93 females) with the same data as the holotype. 17 paratypes are deposited in UB, 8 in PDL and 70 in IAvH.

#### Additional material examined.

95 females with the same data as the holotype.

#### Diagnosis.

See Diagnosis of *Zapatella grahami* above. It also resembles the Nearctic *Callirhytis medularis* Weld (see Discussion).

#### Description

(Figs 19–30). Asexual form.

**Length.** Female 1.7–2.8 mm (n = 50).

**Coloration.** Body, antennae and legs uniformly reddish brown, only tips of mandibles, postocciput, propleura and tarsal claws always darker; in some specimens 3^rd^ and subsequent tergites darker.

**Figures 19–24. F4:**
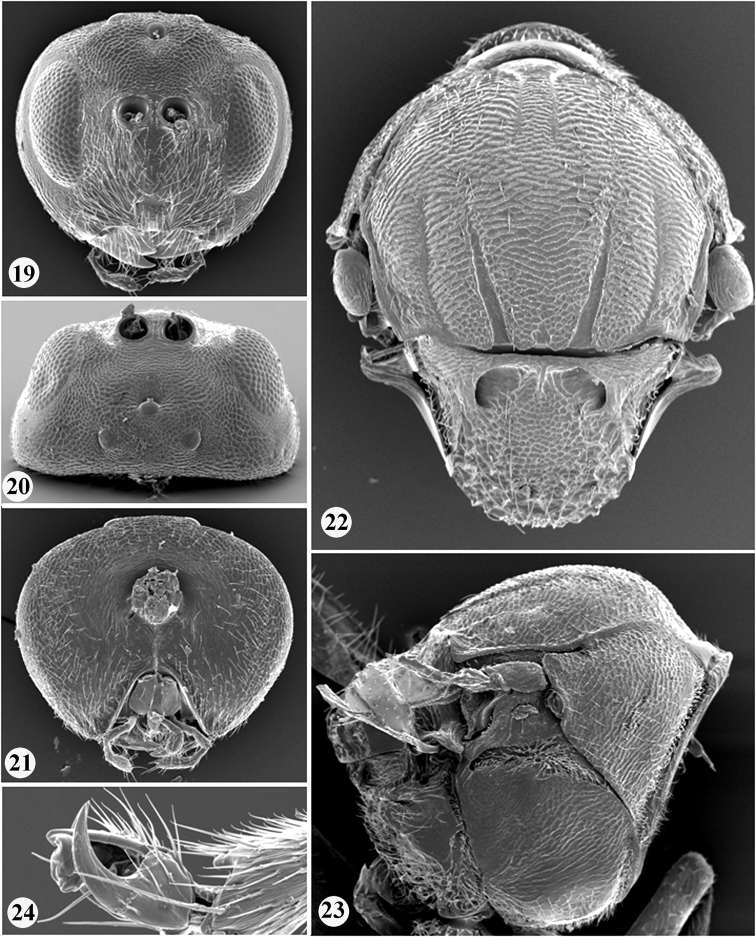
*Zapatella nievesaldreyi*, female **19** head (anterior view) **20** head (dorsal view) **21** head (posterior view) **22** mesosoma (dorsal view) **23** mesosoma (lateral view) **24** tarsal claw.

**Head** (Figs 19–21). Slightly broader than mesosoma, with few white sparse, short inconspicuous setae, more dense on lower face. Head very slightly transverse, only 1.2–1.3 times as broad as high in anterior view and massive from above, only 1.6–1.8 times as broad as long in dorsal view; gena broadened behind eye, broader than transverse diameter of eye, delicately uniformly reticulate; malar space without sulcus, 0.4–0.5 times as long as eye height, with striae radiating from clypeus and nearly reaching eye margin. Lower face delicately coriaceous, without elevated area medially. Clypeus slightly impressed, setose, alutaceous, rounded and slightly emarginate ventrally, medially not incised, anterior tentorial pits small, indistinct; epistomal sulcus and clypeo-pleurostomal line distinct. POL = OOL, OOL 2.5 times as long as length of lateral ocellus and 1.5 times as long as LOL, interocellar area microreticulate, not elevated; frons, vertex and occiput microreticulate; postocciput and postgenae alutaceous. Labial palpus 3-segmented, terminal peg distinct, all three segments densely setose; maxillary palpus 5-segmented, terminal peg distinct, three terminal segments densely setose.

**Figures 25–31. F5:**
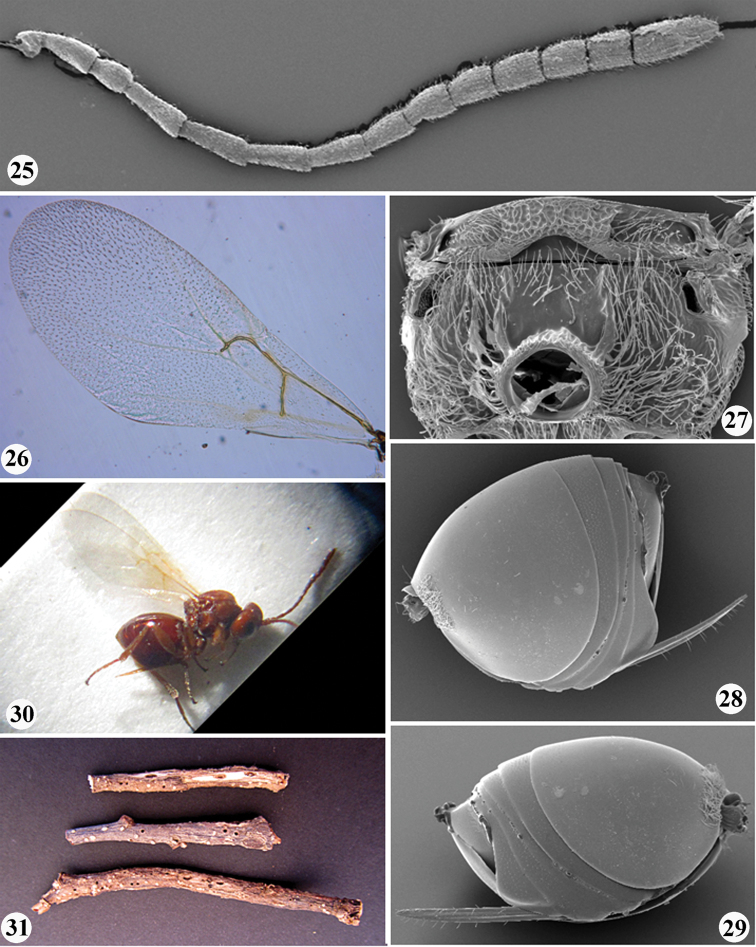
*Zapatella nievesaldreyi*,female: **25** antenna **26** forewing **27** metascutellum and propodeum (posterodorsal view) **28** metasoma (lateral view) **29** metasoma with ventral spine of hypopygium (lateral view) **30** female habitus (lateral view) **31** twigs with galls.

**Antenna** (Fig. 25). 11 flagellomeres, slightly longer than combined length of head and mesosoma; pedicel slightly longer than broad; F1 length nearly equal to length of F2 and slightly longer than F3; F6–F10 shorter and broader than preceding segments; F11 2.0 times as long as F10; placodeal sensilla on F5–F11, hardly traceable or invisible on F1–F4.

**Mesosoma** (Figs 22–23, 27). 1.4 times as long as high, mesoscutum dorsally concave in later view. Pronotum setose, with uniformly delicately reticulate sides, without carinae posterolaterally. Mesoscutum slightly broader than long in dorsal view, with sparse scattered setae; with transverse, delicate interrupted striae which are connected with longitudinally orientated weak striae forming an irregular network of striae, together forming an irregular reticulate surface sculpture. Notauli extending nearly to half length of mesoscutum, deep and broad posteriorly, narrowing toward anterior end, with smooth bottom; median mesoscutal line absent or present in a form of short triangle; parapsidal lines distinct, extending to half length of mesoscutum; anterior parallel lines distinct, extending to 1/3 length of mesoscutum. Mesopleuron uniformly reticulate. Mesoscutellum as broad as long in dorsal view, centrally delicately coriaceous, dull rugose along sides and in posterior 1/3; scutellar foveae transversely ovate, with smooth and shiny bottom, distinctly separated medially by elevated coriaceous area. Metascutellum rugose, higher than height of smooth, shiny ventral impressed area of metanotum; metanotal trough smooth, shiny, with numerous white setae. Propodeum coriaceous, with dense white setae laterally; with smooth, shiny central propodeal area, delimited by distinct parallel lateral carinae, which slightly converge in posterior 1/3; anterior half of central propodeal area with dense white setae, posterior half without setae. Nucha with longitudinal rugae.

**Forewing** (Fig. 26). Nearly as long as body, pubescent, without cilia on margins; radial cell open, around 3.5 times as long as broad; veins very light, hardly traceable; areolet indistinct, usually invisible; vein Rs+M points slightly below midway along basalis; R1 and Rs never reach wing margin, very inconspicuous, often invisible or absent.

**Legs** (Fig. 24). Tarsal claws simple, without basal lobe, but with broad base; hind coxae with dense white setae dorsoposteriorly.

**Metasoma** (Figs 28–29). As long as head and mesosoma together, slightly longer than high; all metasomal tergites smooth and shiny; base of 2nd metasomal tergite with felt-like dense ring of white setae, interrupted dorsally, and a few scattered setae on lateral surface of tergite. Narrow posterior band on 2^nd^ metasomal tergite and all subsequent tergites with very delicate, dense micropunctures. Prominent part of ventral spine of hypopygium needle-like, tapering to apex, 6.0–7.0 times as long as broad, with two parallel rows of short, white, scattered setae.

**Gall** (Fig. 31). Inconspicuous galls in twigs, without visible enlargement (swelling) of the infested twig (branch). The larval cells, 2×1 mm, are nested in the wood parallel one to another.

#### Biology.

Only females are known to induce galls hidden in twigs on *Quercus humboldtii*. Twigs with galls were collected in May and adult wasps immediately emerged in the same month.

#### Distribution.

Currently known only from Colombia, Boyaca, from deciduous mixed broad-leaved forests located about 2000 m altitude.

#### Etymology.

In recognition of the continuing contribution of Dr. José Luis Nieves-Aldrey (Museo Nacional de Ciencias Naturales-CSIC, Departamento de Biodiversidad y Biología Evolutiva, Madrid, Spain) to research on oak gallwasps.

#### Species transferred to *Zapatella*.

Five Nearctic *Callirhytis* species possess the same character set as the above two species and thus they are transferred to *Zapatella*.

### Zapatella cryptica

(Weld) comb. n.

http://species-id.net/wiki/Zapatella_cryptica

**Figures 32–38. F6:**
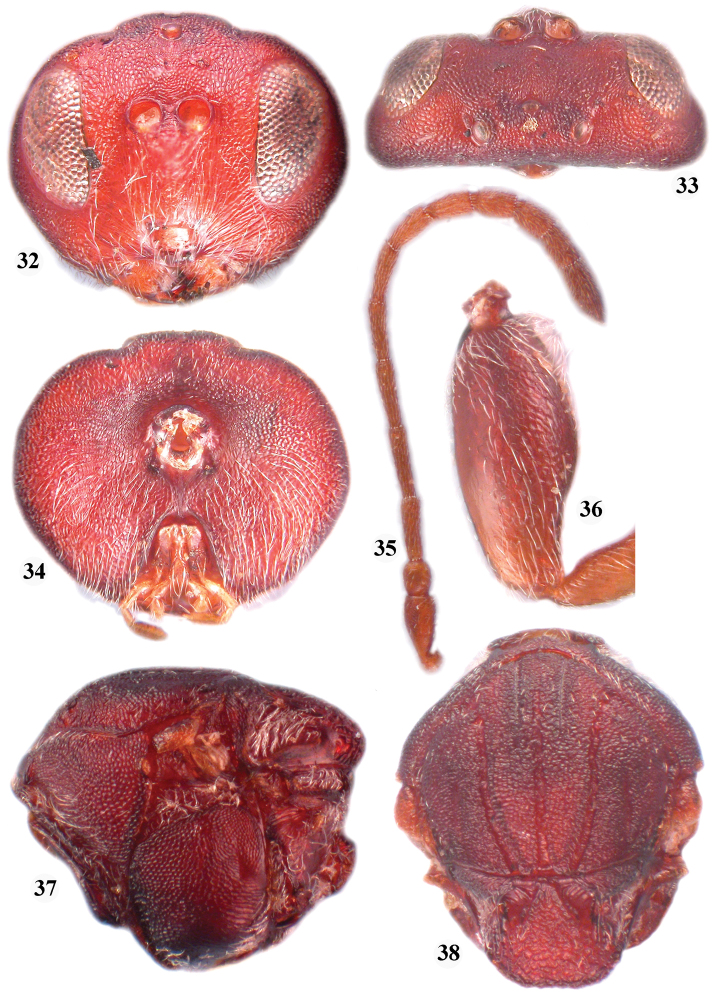
*Zapatella cryptica*, female **32** head (anterior view) **33** head (dorsal view) **34** head (posterior view) **35** antenna **36** hind coxa **37** mesosoma (lateral view) **38** mesosoma (dorsal view).

Figures 32–38 59, 61

Callirhytis cryptica Weld, 1922b (female, galls).

#### Material examined.

One paratype female: ‘Dolhan, Ala, May; *Quercus digitata*; 1188; Paratype No. 24725 USNM’.

Only the asexual generation is known. It induces bud galls on *Quercus myrtifolia* Willd. and *Quercus falcata* Michx. in the USA (Florida and Alabama) (Weld 1922b, Burks 1979).

Type galls were collected in October and adults emerged the next year in May (Weld 1922). The affected terminal bud cluster becomes enlarged, one or two green leaves sometimes grow out beyond the bud scales, and later the bud turns brown; the gall is completely hidden within the bud and is conical, with a thin-walled cell and a tuft of hairs near the apex (Weld 1922b).

The female is entirely uniformly reddish brown and the notauli are incomplete, reaching to 3/4 of the mesoscutum length, but darker lines that look like notauli reach the anterior margin of the mesoscutum; the median mesoscutal line is impressed and reaches the pronotum; the prominent part of the ventral spine of the hypopygium is 6.3 times as long as broad. See also the *Zapatella* species key.

### Zapatella herberti

(Weld) comb. n.

http://species-id.net/wiki/Zapatella_herberti

Figures 39–45

Eumayria herberti Weld, 1926 (females, galls). *Bassettia herberti* (Weld) (Burks 1979). *Callirhytis herberti* (Weld) (Melika and Abrahamson 2007).

#### Material examined.

Two paratype females: ‘Placerville Cal., May 21’18; 1615; Paratype No. 27223 USNM; *Eumayria herberti*’ and ‘Placerville Cal.; cut out May 13; 1615; Paratype No. 27223 USNM; *Eumayria herberti*’.

Only the asexual generation is known. It induces stem swelling galls on *Quercus agrifolia* Née, *Quercus kellogii* Newb., *Quercus wislizeni* A. DC in California (USA) (Weld 1926, Burks 1979). Larval chambers (cells) are nested in the peripheral layer of wood, just under the bark of vigorous shoots 2cm or less in diameter. Adults emerge in late May (Weld 1926).

The female is unifromly reddish brown, including the metasoma. The notauli are complete, always reaching pronotum, deeply impressed; the median mesoscutal line is short, extending to 1/4 of the mesoscutum length, beyond which it is indicated by a dark line only. The metasoma has a ring of very dense white setae at the base of the 2nd metasomal tergite, interrupted dorsally; the metasoma is slightly higher than long in lateral view. The 2nd metasomal tergite is smooth, shiny, without pnctures, while the next tergites have micropunctures. The ventral spine of the hypopygium is hidden under the tergites, its prominent part 6.1 times as long as broad ventrally. See also the key to *Zapatella* species.

**Figures 39–45. F7:**
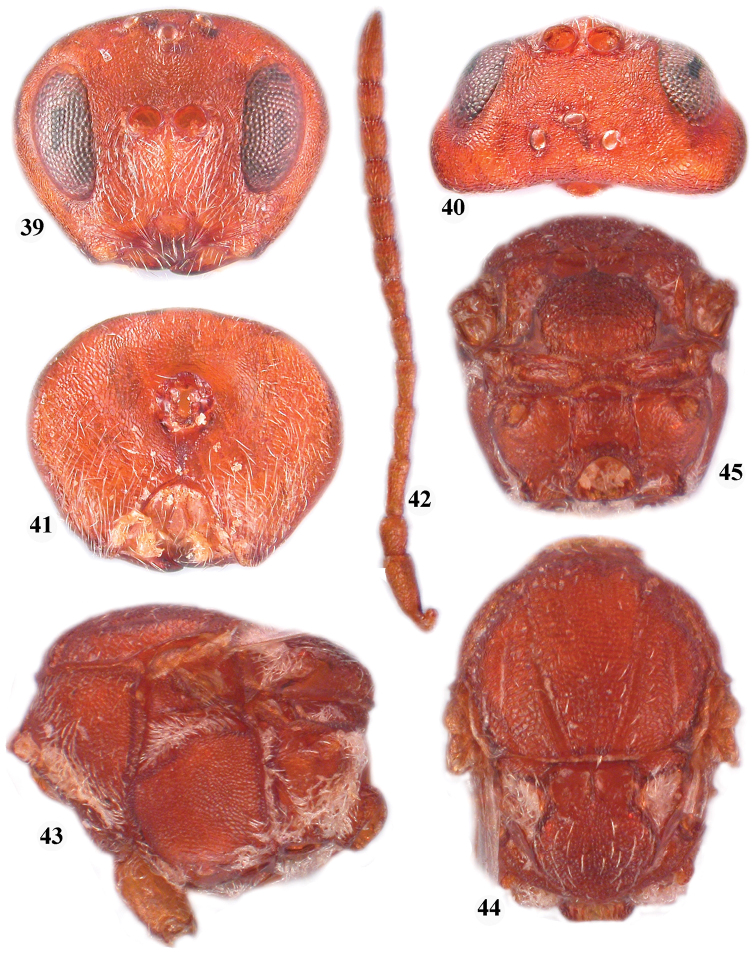
*Zapatella herberti*, female **39**, head (anterior view) **40** head (dorsal view) **41** head (posterior view) **42** antenna **43** mesosoma (lateral view) **44** mesosoma (dorsal view) **45** metascutellum and propodeum (posterodorsal view).

### Zapatella quercusmedullae

(Ashmead) comb. n.

http://species-id.net/wiki/Zapatella_quercusmedullae

Figures 46–51

Cynips quercusmedullae Ashmead, 1885 (females, galls). *Andricus (Andricus) medullae* Ashmead, 1885. *Callirhytis quercusmedullae* (Ashmead) (Burks 1979). *Andricus cryptus* Ashmead, 1887 (synonym in Dailey and Menke 1980).

#### Material examined.

One paratype female: ‘Jacksonville; collector Ashmead; Paratype No. 1497; *Andricus medullae* Ashm. (handwritten label)’.

Only the asexual generation is known. It induces stem swelling galls, in spring, on *Quercus incana* Bartram (= *Quercus cinerea* Raf.), *Quercus marilandica* (L.) Münchn. and *Quercus myrtifolia* in the USA (Florida, Alabama, Georgia, Missisipi, Texas) (Burks 1979). The adults emerge the following year in February and March (Ashmead 1885a, b; Weld 1959).

The female, like the previous species, has the notauli incomplete, extending to half of the mesoscutum length, with darker lines that resemble notauli reaching the anterior margin of the mesoscutum. The median mesoscutal line is absent. The prominent part of the ventral spine of the hypopygium is 6.2 times as long as broad ventrally. See also the key to *Zapatella* species.

**Figures 46–51. F8:**
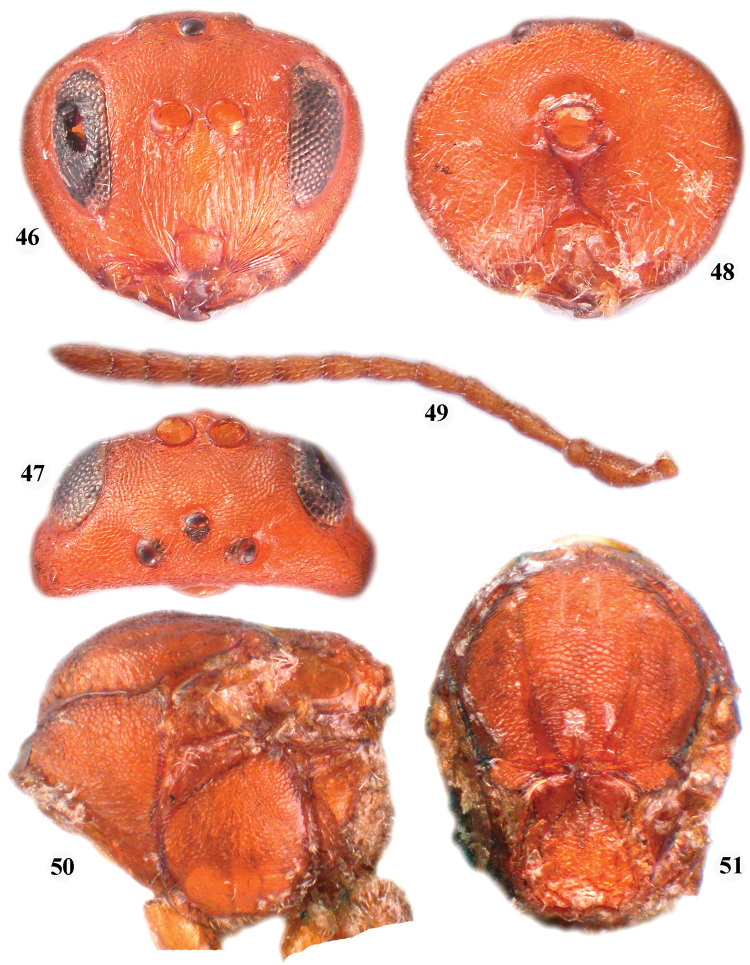
*Zapatella quercusmedullae*, female **46** head (anterior view) **47** head (dorsal view) **48**  head (posterior view) **49** antenna **50** mesosoma (lateral view) **51** mesosoma (dorsal view).

### Zapatella quercusphellos

(Osten Sacken) comb. n.

http://species-id.net/wiki/Zapatella_quercusphellos

**Figures 52–58. F9:**
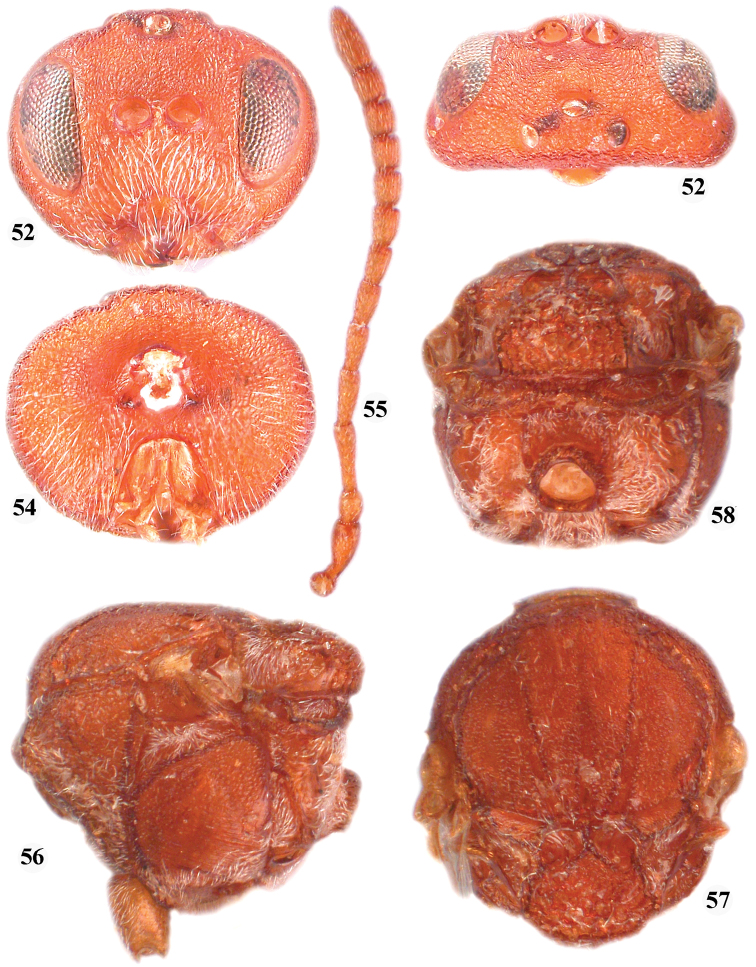
*Zapatella quercusphellos*, female **52** head (anterior view) **53** head (dorsal view) **54** head (posterior view) **55** antenna **56** mesosoma (lateral view) **57** mesosoma (dorsal view) **58** metascutellum and propodeum (posterodorsal view).

Figures 52–58 60, 62

Cynips quercusphellos Osten Sacken, 1861. *Callirhytis quercusphellos* (Osten Sacken ) (Burks 1979).
Cynips quercussimilis Bassett, 1864, syn. n. *Callirhytis quercussimilis* (Bassett) (Burks 1979).

#### Material examined.

**For *Cynips quercusphellos*:** One paratype female:Osten-Sacken coll.; Type (red); Paratype 24684, USNM (red); Cynips quercus-phellos OS from M.C.Z 1921 exchange. One female: Mnt Vernon, Va., 16, 1916, WLMaCtee collector. Callirhytis phellos (OS) det. Weld, 1942 (Weld’s handwriting labels); other female: Alachua Co., Fl., Gainesville, III.27.1924. T.H.Hubbell. Callirhytis quercusphellos (OS) det. Weld, 1925 (Weld’s handwriting label). The two specimens were compared by GM to the Osten Sacken’s cotype, deposited at the USNM (Washington, DC) and obtained by L.H. Weld from the Museum of Comparative Zoology by exchange (Weld 1922b) and they appeared to be identical with that specimen (cotype). **For *Cynips quercussimilis*:** Three female and one male paratypes: ‘Waterbury, Ct., H.F.Bassett Coll.; Type; Beut. Coll rec’d 1935’.

*Callirhytis (Cynips) quercussimilis* (sexual form) was known, inducing stem swelling galls on *Quercus incana*, *Quercus falcata*, *Quercus ilicifolia* Wangenh., *Quercus imbricaria* Michx., and *Quercus myrtifolia* along the Atlantic coast, from Florida to New York state (Burks 1979). The galls are club-shaped swellings if they form on terminal twigs, with 1–4 cells (Weld 1959). Green and fleshy galls develop in May, and later turn woody (become lignified). Adults emerge in June to the beginning of July (Bassett 1864, Weld 1959).

The author of *Callirhytis quercusphellos* (Osten Sacken 1861) collected greenish rounded woody swellings at the tip of the twigs of *Quercus phellos* (L.) in Virginia, near Potomac river in June; four sexual females emerged by the end of June Osten Sacken (1865) mentioned that his species somehow resembles *Callirhytis quercussimilis* (Bassett), however, differs from it. Dalla Torre and Kieffer (1910) treated them as different species. Weld (1922b) erroneously synonymised *Cynips similis* Bassett to *Callirhytis quercusphellos* (O.S.). Later, the two species were treated as different species (Weld 1926, 1928, 1951, 1959; Burks 1979). Weld (1922b) observed galls absolutely similar to those of *Zapatella quercusphellos* on *Quercus falcata*, *Quercus incana*, *Quercus texana* Buckley, *Quercus laurifolia* Michx. and *Quercus myrtifolia*.

*Zapatella quercusphellos* was collected also at Rosslyn, Virginia from *Quercus imbricaria* in June and *Quercus phellos* in May, adults emerged in late June. In both cases the greenish fresh galls were similar terminal enlargements on new growths, inconspicuous, only 5 mm long; after maturation galls were 8–10 mm in diameter (Weld 1926).

A detail examination of specimens of *Callirhytis quercusphellos* and *Callirhytis quercussimilis*, mentioned above, showed no appreciable morphological differences and thus, *Callirhytis quercussimilis* is a syn. n. of *Callirhytis quercusphellos* and here in the species transferred to the *Zapatella* genus, *Zapatella quercusphellos*, comb. n. Females are uniformly dark reddish brown; the notauli are incomplete, extending to half the mesoscutum length, with darker lines reaching the anterior margin of the mesoscutum; the median mesoscutal line extending to 1/2 of the mesoscutum length, further indicated by a dark line only; the prominent part of the ventral spine of the hypopygium is 6.2 times as long as broad ventrally. The male is much darker than the female, with a dark brown head and mesosoma, while the metasoma is slightly lighter (otherwise quite similar to *Zapatella grahami*). See also the key to *Zapatella* species.

Only the sexual generation is known. It induces stem swelling galls on *Quercus incana*, *Quercus falcata*, *Quercus ilicifolia* Wangenh., *Quercus imbricaria*, *Quercus myrtifolia* and *Quercus phellos* along the Atlantic coast, from Florida to New York state (Burks 1979).

**Figures 59–62.**
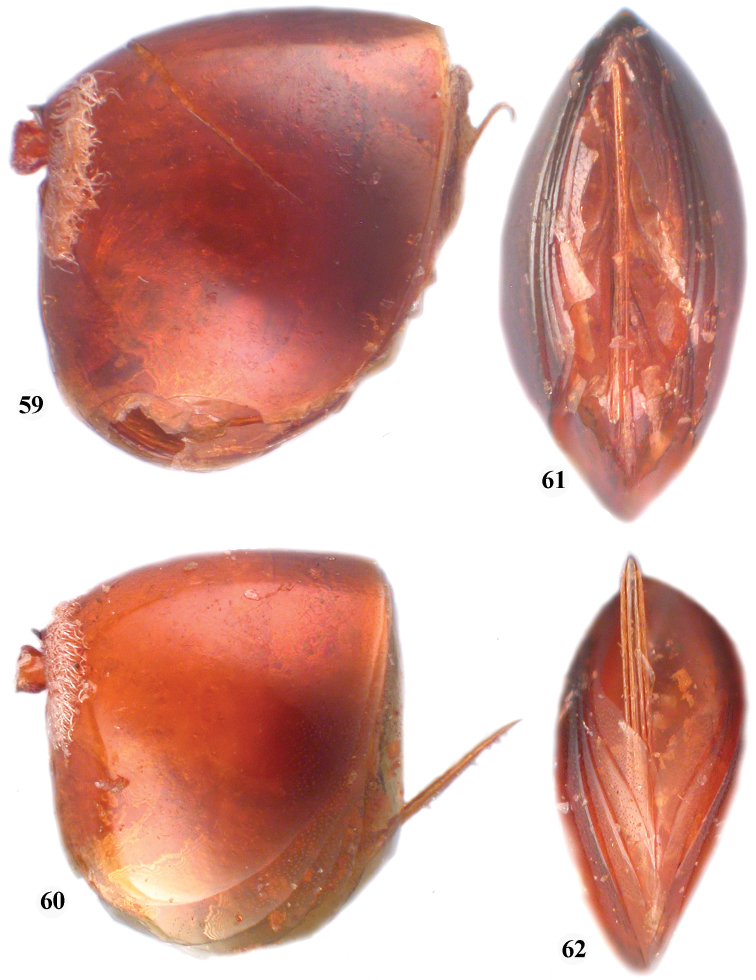
**59** *Zapatella cryptica*, metasoma, female (lateral view) **60** *Zapatella quercussimilis*, metasoma, female (lateral view) **61** *Zapatella cryptica*, ventral spine of hypopygium **62** *Zapatella quercussimilis*, ventral spine of hypopygium.

### Zapatella oblata

(Weld) comb. n.

http://species-id.net/wiki/Zapatella_oblata

Figures 63 71

Callirhytis oblata Weld, 1952.

#### Material examined.

Paratype female: Vienna, Va., March 21’46. Q. coccinea, 558, Paratype 60128, Callirhytis oblata Weld.

Only the asexual generation is known. It induces bud galls on *Quercus coccinea* Muench. and *Quercus falcata* in Virginia, USA (Weld 1952). The frons, vertex and head posteriorly are dark brown to black, the mesoscutum along and between anterior parallel lines and along parapsidal lines is black, scutellar foveae and the central propodeal area are also dark brown; the rest of the body is reddish brown. Notauli are complete, the median mesoscutal line extending to 2/3 of the mesoscutum length, scutellar foveae transverse; the prominent part of the ventral spine of the hypopygium is very long, about 8.5 times as long as broad from ventral view.

**Figures 63–68.**
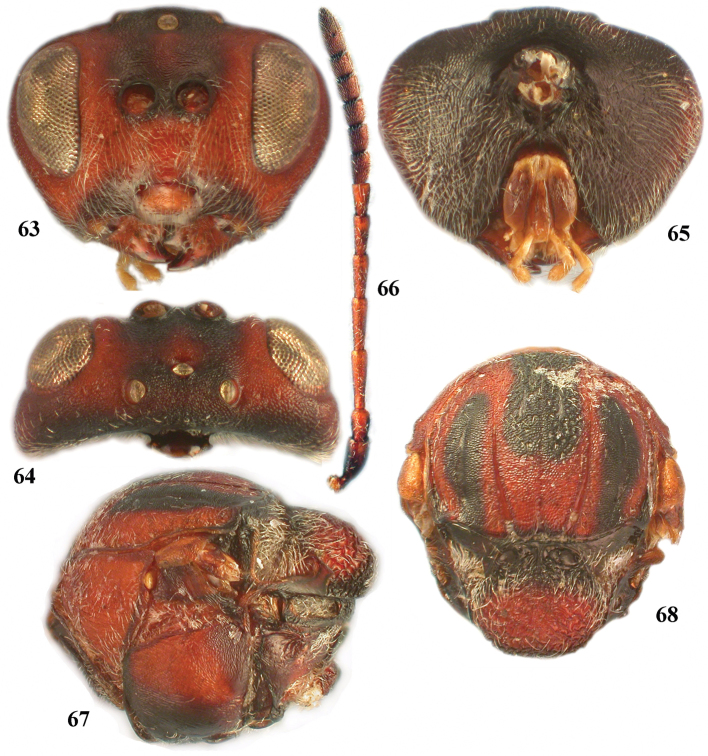
*Zapatella oblata*, female **63** head (anterior view) **64** head (dorsal view) **65** head (posterior view) **66** antenna **67** mesosoma (lateral view) **68** mesosoma, (dorsal view).

**Figures 69–71.**
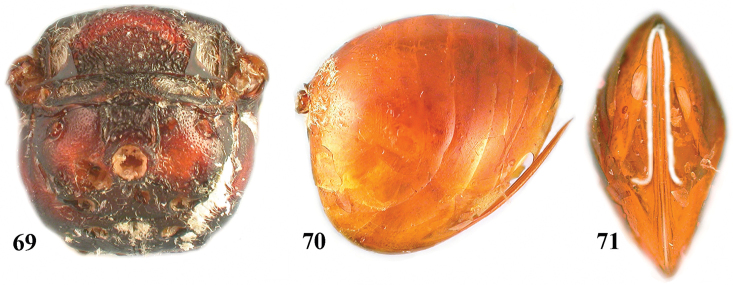
*Zapatella oblata*, female **69** metascutellum and propodeum (posterodorsal view) **70** metasoma (lateral view) **71** ventral spine of hypopygium (ventral view).

### Key to *Zapatella* species

**Table:** 

1	Female, antenna with 11 flagellomeres	2
–	Male, antenna with 13 flagellomeres	8
2	Median mesoscutal line extending to 1/3–2/3 of mesoscutum length, deeply impressed (Figs 38, 57)	3
–	Median mesoscutal line very short or absent (Figs 10, 22, 51)	5
3	Median mesoscutal line impressed to 1/2 of mesoscutum length, indicated beyond this by dark line; prominent part of ventral spine of hypopygium 6.2 times as long as broad in ventral view (Figs 60, 62). Stem swelling galls	*Zapatella quercusphellos*
–	Median mesoscutal line extending to 2/3 of mesoscutum length; prominent part of ventral spine of hypopygium at least 8.0–8.5 times as long as broad in ventral view (Figs 59, 61, 71). Bud galls	4
4	Head and mesosoma uniformly reddish brown (Figs 32–38); scutellar foveae quadrangular, as long as broad (Fig. 38)	*Zapatella cryptica*
–	Head and mesosoma with large dark brown to black patches (Figs 63–68); scutellar foveae transverse, broader than high (Fig. 68)	*Zapatella oblata*
5	Body darker, head and mesosoma always with large dark brown to black spots (Figs 1, 2, 4, 5, 9, 10, 12); POL 1.4 times as broad as OOL (Fig. 2); bottom of scutellar foveae with rugae (Fig. 11); prominent part of ventral spine of hypopygium 7.5-8.5 times as long as broad (Figs 14, 16). Acorn galls (Figs 17–18)	*Zapatella grahami*
–	Body entirely and uniformly light reddish brown, without or with very few darker spots; POL equal to OOL (Fig. 20, 40); bottom of scutellar foveae smooth, without rugae (Fig. 22, 44); prominent part of ventral spine of hypopygium 6.0–7.0 times as long as broad (Figs 29–30). Galls in twigs	6
6	Notauli complete, always reaching pronotum, deeply impressed (Fig. 44). Stem swelling galls with larval chambers nested in the peripheral layer of wood	*Zapatella herberti*
–	Notauli incomplete, extending to 1/2 of mesoscutum length, never reaching pronotum (Figs 22, 51). Galls in twigs with larval chambers nested in the wood	7
7	Head in anterior view ovate (Fig 46), less robust and transverse from above (Fig. 47); scutellar foveae separated by very thin, line-like median carina (Fig. 51); lateral propodeal carinae subparallel, extending the entire length (as in Fig. 58). Stem swelling galls	*Zapatella quercusmedullae*
–	Head in anterior view rounded (Fig 19), robust and less transverse (Fig 20); scutellar foveae separated by broad bar (Fig 51); lateral propodeal carinae slightly bent outwards in posterior 1/3 (Fig. 27). Inconspicuous galls in twigs (Fig. 31)	*Zapatella nievesaldreyi*
8	Gena width nearly equal to transverse diameter of eye; scutellar foveae not separated by median carina, not delimited posteriorly, with reticulate bottom. Acorn galls (Figs 17–18)	*Zapatella grahami*
–	Gena width no more than 1/3 of transverse diameter of eye; scutellar foveae separated by distinct median carina, well-delimited all around, with smooth shiny bottom. Stem swelling galls	*Zapatella quercusphellos*

## Discussion

Although the newly described genus *Zapatella*, somewhat resembles *Bassettia*, *Loxaulus*, and *Plagiotrochus* (see Diagnosis to *Zapatella* and Table 1), it most closely resembles *Callirhytis* ‘sensu lato’ (Weld 1952). The genus *Callirhytis*, with morphological generic limits given by Weld (1952), and widely used by many researchers over many decades, is a problematic one. Originally *Callirhytis* was erected for the European species by Förster (1869) and the main generic diagnostic characters he proposed were the transversely striate mesoscutum and the presence of the malar sulcus. Later, several authors (Mayr 1881, Ashmead 1885a, Cameron 1893, Kieffer 1897-1901; among others) considered it as a subgenus of *Andricus*, where they placed species with simple tarsal claws, while the subgenus *Andricus* (*Andricus*) included species in which the tarsal claws possess a basal lobe (toothed tarsal claw). Mayr (1902) restored the generic status of *Callirhytis*. Nieves-Aldrey (1992), in his revision of the European species, showed that different *Callirhytis* species, in different alternate generations, vary in the presence or absence of toothed tarsal claws. Weld (1922a, 1922b, 1926, 1952) included many species in *Callirhytis* and established particular species groups, but neglected the diagnostic characters of *Callirhytis* given by Förster (1869), and as the result, the concept of *Callirhytis* became very chaotic. As a consequence, the Nearctic *Callirhytis* is a polyphyletic group what was already shown earlier (Nylander 2004, Liljeblad et al. 2008) and many North American species assigned to *Callirhytis* (Weld 1952, Burks 1979), in fact, are not *Callirhytis* ‘sensu stricto’ (Melika and Abrahamson 2002, Melika et al. 2009a). Seven species of *Callirhytis* ‘sensu stricto’are known from the Western Palaearctic Region (Melika 2006).

**Table 1.** Generic characteristics of *Zapatella* and allied genera (exclusive generic characters of *Zapatella* are in bold)

Characters	*Loxaulus*	*Bassettia*	*Plagiotrochus*	*Callirhytis*	*Zapatella*
Malar sulcus	present	absent	absent	present	absent
Mesoscutum sculpture	reticulate	reticulate	transversely coriaceous or rugose	transversely strongly carinated	reticulate
Pronotum sculpture	reticulate	reticulate	fine striae	strong striae	reticulate
Metascutellum sculpture	rugoso-reticulate	reticulate	rugoso-reticulate	rugose	rugoso-reticulate
Metanotal trough	glabrous	glabrous	glabrous	glabrous	**pubescent**
Ventral spine	short (<2.5)	short (<2.0)	short (<3.0)	short (<4.0)	**long (>6.0)**
Head fom above	massive	massive	oblong/lunate	oblong/lunate	massive
Sculpture of mesopleuron	uniformly reticulate	uniformly reticulate	with transverse sculptured band only	glabrous or coriaceous	with transverse sculptured band only
Propodeal carinae	subparallel	subparallel	strongly bent outwards	subparallel	subparallel
Hind coxae dorsoposterior surface	glabrous	glabrous	glabrous	glabrous	**pubescent**
Forewing margin	variable	no cilia	with cilia	no cilia	no cilia
F1 in male incised	yes	no	yes	yes	yes
R1 and Rs veins	conspicuous	conspicuous	conspicuous	conspicuous	**inconspicuous**
Scutellar foveae	anterior impression, not separated	present, posteriorly undefined	present, posteriorly undefined	present, posteriorly undefined	present, well-delimited posteriorly
2nd metasomal tergite, lateral setae	glabrous, few setae	glabrous, few setae	glabrous, few setae	glabrous, few setae	**with dense ring of setae**
3rd metasomal tergite	smooth	smooth	smooth	smooth	**punctate**

Melika and Abrahamson (2002) stated that of the 115 described Nearctic *Callirhytis* species (Burks 1979), only 15 are true *Callirhytis* ‘sensu stricto’: *Callirhytis cedrosensis* Dailey & Sprenger, *Callirhytis corrugis* (Bassett) (= *defecta* Kinsey), *Callirhytis eldoradensis* (Beutenmueller), *Callirhytis electrea* Weld, *Callirhytis flora* Weld (= *Callirhytis milleri*), *Callirhytis fructicola* Ashmead, *Callirhytis fructuosa* Weld, *Callirhytis intersita* Weld, *Callirhytis lapillula* Weld, *Callirhytis medularis* Weld, *Callirhytis morrisoni* (Ashmead), *Callirhytis perrugosa* Weld,* C. petrina* Weld, *Callirhytis petrosa* Weld, and *Callirhytis quercusmedullae* (Ashmead). Melika & Abrahamson (2002) also transferred some *Andricus* and *Bassettia* species to *Callirhytis* ’sensu stricto’: *Callirhytis albobalani* (Weld), *Callirhytis chrysobalani* (Weld), *Callirhytis coortus* (Weld), *Callirhytis coronus* (Beutenmueller), *Callirhytis montezuma* (Beutenmueller), *Callirhytis rhizoxenus* Ashmead, *Callirhytis wheeleri* (Beutenmueller), *Callirhytis ceropteroides* Bassett, and *Callirhytis herberti* (Weld). In this study, *Callirhytis quercusmedullae* and *Callirhytis herberti*, are transferred to *Zapatella* genus, and probably some other listed species also form different monophyletic groups.

The newly established *Zapatella*, with the two described neotropic species and five transferred *Callirhytis* species, is the first contribution to this ‘reorganization’ of the Nearctic *Callirhytis* ‘sensu lato’. Some *Callirhytis* species (*Callirhytis balanaspis* Weld, *Callirhytis corrugis* (Bassett), *Callirhytis glomerosa* Weld, and *Callirhytis medularis* Weld) partially resemble *Zapatella* in their host plant associations, morphology of adults and/or galls they induce, and thus need some explanation.

*Callirhytis balanaspis* Weld (only the asexual generation is known) induces acorn galls on red oaks, also has a very pale venation, R1 invisible, the malar sulcus absent, the mesoscutum with delicate, net-like reticulate transverse sculpture, as in *Zapatella*. However, the ring of very dense white setae at the base of the 2^nd^ metasomal tergite is absent and the prominent part of the ventral spine of the hypopygium is only 3.0–3.5 times as long as broad. This species is definitely not a *Callirhytis* ‘sensu stricto’, it closely resembles *Zapatella*, and form a discrete unit within *Callirhytis* ‘sensu lato’.

In *Callirhytis corrugis* (Bassett), which induces acorn galls on red oaks (Burks 1979), the forewing venation is pale, with some veins invisible, but the mesoscutum is coarsely transversely rugose, the malar sulcus is present, POL is shorter than OOL, the female antenna has 12 flagellomeres, the ring of very dense white setae at the base of the 2^nd^ metasomal tergite is absent, and the prominent part of the ventral spine of the hypopygium is less than 2.0 times as long as broad. It is a true *Callirhytis* as noted in Melika and Abrahamson (2002).

In *Callirhytis glomerosa* Weld, which induces bud galls on red oaks, the malar sulcus is absent, the ring of very dense white setae at the base of the 2^nd^ metasomal tergite is present, the hind coxae have dense white setae on the dorsoposterior surface as in *Zapatella*; the mesoscutum is very finely transversely rugose and the prominent part of the ventral spine of the hypopygium is much shorter, as in *Callirhytis*. However, it differs form both genera in the trapezoid head in anterior view (much shorter from above) and the female antenna with 12 flagellomeres. This species is definitely not a *Callirhytis* ‘sensu stricto’, closely resembles *Zapatella*, and forms a discrete unit within *Callirhytis* ‘sensu lato’.

*Callirhytis medularis* Weld induces stem swelling galls on red oaks and is only known from the sexual generation. However, in *Callirhytis medularis*, the female antenna has 12 flagellomeres, the head is more massive from above, much broader than the mesosoma; the mesoscutum is dull rugose, with strong transverse ridges, the mesoscutellum broader than long, the metanotal troughs and hind coxae without dense white setae, the 2nd metasomal tergite without a ring of dense white setae at the base; the ventral spine of the hypopygium is much shorter. This species is definitely not a *Callirhytis* ‘sensu stricto’, closely resembles *Zapatella*, and forms a discrete unit within *Callirhytis* ‘sensu lato’.

Preliminary morphological analysis (GM and JPV, *umpublished data*) also showed that some Nearctic *Callirhytis* species that induce stem swelling galls on different sections of oaks, form distinct morphological and phylogenetic units. Thus, some of these discrete morphological groups form distinct genera, which might be monophyletic groups.

## Supplementary Material

XML Treatment for Zapatella

XML Treatment for Zapatella grahami

XML Treatment for Zapatella nievesaldreyi

XML Treatment for Zapatella cryptica

XML Treatment for Zapatella herberti

XML Treatment for Zapatella quercusmedullae

XML Treatment for Zapatella quercusphellos

XML Treatment for Zapatella oblata

## References

[B1] Ashmead WH (1885a) On the cynipidous galls of Florida with descriptions of new species. Transactions of the American Entomological Society 12: 5-9.

[B2] Ashmead WH (1885b) Studies in North American Chalcididae, with descriptions of new species from Florida. Transactions of the American Entomological Society 12: 11-19.

[B3] Bassett HF (1864) Descriptions of several new species of Cynips and a new species of *Diastrophus*. Proceedings of the Entomological Society of Philadelphia 3: 679-691.

[B4] Benia F Khelil M Pujade-Villar J (2009) *Plagiotrochus amenti*, una espèce gallicole potentiellement dangereuse pour la chêne-liége (*Quercus suber* L.) trouvée pour la première fois en Algérie (Hymenoptera, Cynipidae). Nouvelle Revue d’Entomologie 25 (4): 291-296.

[B5] Burks BD (1979) Superfamily Cynipoidea. In: Krombein KV Hurd PD Jr Smith DR Burks BD (Eds). Catalog of Hymenoptera in America North of Mexico. Vol. 1. Symphyta and Apocrita. Smithsonian Institution Press, Washington, DC: 1045-1107.

[B6] Cameron P (1883) Zoology. Insecta. Hymenoptera. Biologia Centrali-Americana 1: 1-497.

[B7] Camacho M Orozco L (1998) Patrones fenológicos de doce especies arbóreas del bosque montano de la Cordillera de Talamanca, Costa Rica. Revista de Biología Tropical 46 (3): 533-542.

[B8] Dailey DC Menke AS (1980) Nomenclatorial notes on North American Cynipidae (Hymenoptera). The Pan-Pacific Entomologist 56 (3): 170-174.

[B9] Dalla Torre KW Kieffer JJ (1910) Cynipidae. Das Tierreich, 24. Berlin, Friedlander & Sohn, 891 pp.

[B10] Fergusson NDM (1995) The cynipoid families. In: Hanson PE Gauld ID (Eds). The Hymenoptera of Costa Rica. Oxford, New York, Tokyo, Oxford University Press: 247-265.

[B11] Folliot R (1961) Sur le regroupement des espèces *Andricus quadrilineatus* Hartig et *Andricus marginalis* Schlechtendal. Comptes Rendus des Sciences de l’Académie des Sciences 253: 3050-3052.

[B12] Folliot R (1964) Contributions a l’étude de la biologie des Cynipides gallicoles (Hymenoptera, Cynipoidea). Annales des Sciences Naturelles, Zoologie 6: 409-564.

[B13] Förster A (1869) Ueber die Gallwespen. Verhandlungen der Zoologische-botanische Gesselschaft Wien 19: 327-370.

[B14] Garbín L Díaz NB Pujade-Villar J (2008) Experimental study of the reproductive cycle of *Plagiotrochus amenti* Kieffer, 1901 (Hymenoptera, Cynipoidea, Cynipidae), with comments on its taxonomy. Boletín de la Asociación Española de Entomología 32(3/4): 341–349.

[B15] Govaerts R Frodin DG (1998) World Checklist and Bibliography of Fagales. Royal Botanic Gardens, Kew, 408 pp.

[B16] Harris R (1979) A glossary of surface sculpturing. State of California, Department of Food and Agriculture, Occasional Papers in Entomology 28: 1-31.

[B17] Kieffer JJ (1897–1901) Monographie des Cynipides d.Europe et d.Algerie. Ibalynae et Cynipinae. Libbairie Scientifique A. Hermann, Paris.

[B18] Kinsey AC (1936) The origin of the higher categories in Cynips. Indiana University, Publication of Science Series 4: 1-334.

[B19] Kinsey AC (1937) New Mexican gall wasps (Hymenoptera, Cynipidae). II. Revista de Entomologia 7: 428-471.

[B20] Liljeblad J Ronquist F (1998) A phylogenetic analysis of higher-level gall wasp relationships (Hymenoptera: Cynipidae). Systematic Entomology 23: 229-252. doi: 10.1046/j.1365-3113.1998.00053.x

[B21] Liljeblad J Ronquist F Nieves-Aldrey J-L Fontal-Cazalla F Ros-Farré P Gaitros D Pujade-Villar J (2008) A fully web-illustrated morphological phylogenetic study of relationships among oak gall wasps and their closest relatives (Hymenoptera: Cynipidae). Zootaxa 1796: 1-73.

[B22] Mayr G (1881) Die Genera der gallenbewohnenden Cynipiden. Jahresberichte der Communal-Oberrealschule im I. Bezirke, Wien 20: 1-38.

[B23] Mayr G (1902) Ueber Nordamerikanische Cynipiden. Verhandlungen der k.k. zoologisch-botanischen Gesellschaft in Wien 52: 287-290.

[B24] Medianero E Nieves-Aldrey JL (2010a) The genus *Amphibolips* Reinhard (Hymenoptera: Cynipidae: Cynipini) in the Neotropics, with description of three new species from Panama. Zootaxa 2360: 47-62.

[B25] Medianero E Nieves-Aldrey JL (2010b) Description of the first Neotropical species of *Bassettia* Ashmead (Hymenoptera: Cynipidae: Cynipini) from Panama. Graellsia 66 (2): 213-220.

[B26] Medianero E Nieves-Aldrey JL (2011a) First record of the genus *Disholcaspis* Dalla Torre & Kieffer (Hymenoptera: Cynipidae: Cynipini) in the Neotropics, with description of two new species from Panama. Zootaxa 2802: 23-33.

[B27] Medianero E Nieves-Aldrey JL (2011b) Primer estudio de las avispas de las agallas de la República de Panamá, incluyendo una lista actualizada de los cinípidos neotropicales (Hymenoptera, Cynipoidea, Cynipidae). Boletín de la Sociedad Entomológica Aragonesa (SEA) 48: 89-104. doi: 10.3989/graellsia.2010.v66.029

[B28] Medianero E Nieves-Aldrey JL Melika G (2011a) Two new neotropical species of oak gall wasps of the genus *Loxaulus* Mayr (Hymenoptera: Cynipidae: Cynipini) from Panama. Zootaxa 2811: 37-46.

[B29] Medianero E Nieves-Aldrey JL Pujade-Villar J (2011b) The genus *Odontocynips* Kieffer (Hymenoptera: Cynipidae: Cynipini) in Panama, with redescription of *Cynips championi* Cameron, 1883. Graellsia 67: 35-46. doi: 10.3989/graellsia.2011.v67.033

[B30] Melika G (2006) Gall Wasps of Ukraine. Cynipidae. Vestnik zoologii, supplement 21(1/2): 1–644.

[B31] Melika G Abrahamson WG (2002) Review of the world genera of oak cynipid wasps (Hymenoptera: Cynipidae, Cynipini). In: Melika G Thuróczy C (Eds). Parasitic Wasps: Evolution, Systematics, Biodiversity and Biological Control. Agroinform, Budapest: 150-190.

[B32] Melika G Abrahamson WG (2007) Review of the Nearctic gall wasp species of the genus *Bassettia* Ashmead, 1887, with description of new species (Hymenoptera: Cynipidae: Cynipini). Acta Zoologica Academiae Scientiarum Hungaricae 53 (2): 131-148.

[B33] Melika G Cibrián-Tovar D Cibrián-Llanderal VD Tormos J Pujade-Villar J (2009a) New species of oak gallwasp from Mexico (Hymenoptera: Cynipidae: Cynipini) – a serious pest of *Quercus laurina* (Fagaceae). Dugesiana 16 (2): 67-73.

[B34] Melika G Pérez-Hidalgo N Hanson P Pujade-Villar J (2009b) New species of oak gallwasp from Costa Rica (Hymenoptera: Cynipidae: Cynipini). Dugesiana 16 (1): 35-39.

[B35] Melika G Equihua-Martínez EG Estrada-Venegas D Cibrián-Tovar VD Cibrián-Llanderal VD Pujade-Villar J (2011a) New *Amphibolips* gallwasp species from Mexico (Hymenoptera: Cynipidae). Zootaxa 3105: 47-59.

[B36] Melika G Hanson P Pujade-Villar J (2011b) A new species of *Disholcaspis* Dalla Torre and Kieffer oak gallwasp from Costa Rica (Hymenoptera: Cynipidae: Cynipini). Dugesiana 18 (1): 17-22.

[B37] Nieves-Aldrey JL (1992) Revision of the European species of the genus *Callirhytis* Förster (Hymenoptera, Cynipidae). Graellsia 48: 171-183.

[B38] Nixon K (2006) Global and Neotropical Distribution and Diversity of Oak (genus *Quercus*) and Oak Forests. Ecological Studies 185: 3-13. doi: 10.1007/3-540-28909-7_1

[B39] Nylander JAA (2004) Bayesian Phylogenetics and the evolution of gall wasps. PhD Thesis, Acta Universitatis Upsaliensis, Uppsala.

[B40] Osten Sacken CR von (1861) On the Cynipidae of the North American oaks and their galls. Proceedings of the Entomological Society of Philadelphia 1-3: 47–72.

[B41] Osten Sacken CR von (1865) Contributions to the Natural History of the Cynipidae of the United States and of their Galls. Article 4th. Proceedings of the Entomological Society of Philadelphia 4: 331-380.

[B42] Pujade-Villar J Hanson P (2006) Familia Cynipidae (las avispas cecidógenas). In: Hanson P Gauld IA (Eds). Hymenoptera de la Región Neotropical. Memoirs of the American Entomological Institute 77: 293–302.

[B43] Pujade-Villar J (2009) Description of *Odontocynips hansoni* n. sp., from Costa Rica (Hymenoptera: Cynipidae). Dugesiana 15(2): 79–85. doi: 10.1590/S1519-566X2009000600015

[B44] Pujade-Villar J Equihua-Martínez A Estrada-Venegas EG Chagoyán-García C (2009) Estado de conocimiento de los Cynipini en México (Hymenoptera: Cynipidae), perspectivas de estudio. Neotropical Entomology 38 (6): 809-821. 20098928 10.1590/s1519-566x2009000600015

[B46] Pujade-Villar J Equihua-Martínez A Estrada-Venegas EG Serrano-Muñoz M Lomeli-Flores JR (2011) Una nueva especie mexicana del género *Andricus* con caracteres muy peculiares: *A. georgei* Pujade-Villar n. sp. (Hymenoptera, Cynipidae). Boletín de la Sociedad Entomológica Aragonesa (SEA) 49: 27-32.

[B47] Pujade-Villar J Hanson P Melika G (2012) A new genus of oak gallwasp, *Coffeikokkos* Pujade-Villar & Melika, gen. n., with a description of a new species from Costa Rica (Hymenoptera, Cynipidae). ZooKeys 168: 19-29. doi: 10.3897/zookeys.168.2030 PMC3293441 22423188

[B48] Ronquist F Nordlander G (1989) Skeletal morphology of an archaic cynipoid, *Ibalia rufipes* (Hymenoptera: Ibaliidae). Entomologica Scandinavica, supplement 33: 1-60.

[B49] Stone GN Hernandez-Lopez A Nicholls JA di Pierro E Pujade-Villar J Melika G Cook JM (2009) Extreme host plant conservatism during at least 20 million years of host plant pursuit by oak gallwasps. Evolution 63: 854-869. doi: 10.1111/j.1558-5646.2008.00604.x 19292826

[B50] Weld LH (1922a) Notes on American Gallflies of the family Cynipidae producing galls on acorns, with descriptions of new species. Proceedings of the United States National Museum 61 (19): 1-32. doi: 10.5479/si.00963801.61-2440.1

[B51] Weld LH (1922b) Notes on Cynipid Wasps, with descriptions of new North American Species. Proceedings of the United States National Museum 61 (18): 1-29.

[B52] Weld LH (1926) Field Notes on Gall-inhabiting Cynipid Wasps with descriptions of new species. Proceedings of the United States National Museum 68 (10): 1-131. doi: 10.5479/si.00963801.68-2611.1

[B53] Weld LH (1928) Cynipidae. In: Leonard L (Ed) Insects of New York. 967–974.

[B54] Weld LH (1951) Superfamily Cynipoidea. In: Muesebeck, Krombein, Townes et al. (Eds) Hymenoptera in America north of Mexico. Synoptic Catalogue. US Department of Agriculture. Agricultural Monograph No. 2, 594–654.

[B55] Weld LH (1952) Cynipoidea (Hym.) 1905-1950 being a Supplement to the Dalla Torre and Kieffer monograph the Cynipidae in Das Tierreich, Leiferung 24, 1910 and bringing the systematic literature of the world up to date, including keys to families and subfamilies and list of new generic, specific and variety names. Privately printed, Ann Arbor, Michigan, privately printed, 351 pp.

[B56] Weld LH (1959) Cynipid galls of the Eastern United States. Ann Arbor, Michigan, privately printed, 124 pp.

